# A multidimensional Bayesian IRT method for discovering misconceptions from concept test data

**DOI:** 10.3389/fpsyg.2025.1506320

**Published:** 2025-01-29

**Authors:** Martin Segado, Aaron Adair, John Stewart, Yunfei Ma, Byron Drury, David Pritchard

**Affiliations:** ^1^Department of Mechanical Engineering, Massachusetts Institute of Technology, Cambridge, MA, United States; ^2^Department of Physics, Massachusetts Institute of Technology, Cambridge, MA, United States; ^3^Department of Physics and Astronomy, West Virginia University, Morgantown, WV, United States; ^4^Department of Electrical Engineering and Computer Science, Massachusetts Institute of Technology, Cambridge, MA, United States

**Keywords:** item response theory, student misconceptions, multiple-choice questions, distractor analysis, multidimensional nominal categories model, mean-field variational inference, hierarchical priors

## Abstract

We present an exploratory method for discovering likely misconceptions from multiple-choice concept test data, as well as preliminary evidence that this method recovers known misconceptions from real student responses. Our procedure is based on a Bayesian implementation of the Multidimensional Nominal Categories IRT model (MNCM) combined with standard factor-analytic rotation methods; by analyzing student responses at the level of individual distractors rather than at the level of entire questions, this approach is able to highlight multiple likely misconceptions for subsequent investigation without requiring any manual labeling of test content. We explore the performance of the Bayesian MNCM on synthetic data and find that it is able to recover multidimensional item parameters consistently at achievable sample sizes. These studies demonstrate the method's robustness to overfitting and ability to perform automatic dimensionality assessment and selection. The method also compares favorably to existing IRT software implementing marginal maximum likelihood estimation which we use as a validation benchmark. We then apply our method to approximately 10,000 students' responses to a research-designed concept test: the Force Concept Inventory. In addition to a broad first dimension strongly correlated with overall test score, we discover thirteen additional dimensions which load on smaller sets of distractors; we discuss two as examples, showing that these are consistent with already-known misconceptions in Newtonian mechanics. While work remains to validate our findings, our hope is that future applications of this method could aid in the refinement of existing concept inventories or the development of new ones, enable the discovery of previously-unknown student misconceptions across a variety of disciplines, and—by leveraging the method's ability to quantify the prevalence of particular misconceptions—provide opportunities for targeted instruction at both the individual and classroom level.

## 1 Introduction

Research-designed multiple-choice concept tests commonly have wrong answer choices (“distractors”) that reflect typical incorrect student responses; these are often discerned from research or by first administering items in open-response format and identifying commonalities in the answers. Earlier work with Pérez-Lemonche et al. (2019) showed that even students whose raw score was below chance strongly favored particular incorrect responses on such tests, implying that systematic mental processes rather than random guessing underlie the selection of distractors. Various theories of these mental processes have been proposed, including knowledge in fragments (diSessa, 1993), ontological categories (Chi and Slotta, 1993), mixed ontological categories or models (Adair, 2013), dual process theories (Gette et al., 2018), misconceptions (a better definition is “an alternative hypothesis [to the current paradigm]”), and specific misunderstandings (e.g., “cannot interpret graphs” or inconsistent errors in applying Newton's 3rd law). The present work is based on the misconception/misunderstanding viewpoint, wherein common wrong answers—and the resulting research-determined distractors—often result from and thus encode common student misconceptions or misunderstandings.

When a research-designed test is administered, student misconceptions (whether already known to the researchers or not) manifest as an increased likelihood of a student co-selecting sets of distractors consistent with their (mis)understandings of the domain. The misconceptions may present in differing degrees for different students, with stronger misconceptions leading students to endorse a higher fraction of those distractors consistent with their incorrect belief. Notably, distractors in any one item may reflect distinct misconceptions, either alone or in combination—in a physics question involving a skydiver for example, one distractor might reflect a common misunderstanding about acceleration, another might encode an incorrect mental model of air resistance, and a third might combine elements of both. That is, *misconceptions are encoded at the level of individual response categories, and less at the level of whole items*. Thus discovering them requires an analysis capable of capturing multidimensionality within categories, not only within items.

In this work, we present an exploratory analysis procedure for discovering the types of misconceptions discussed above. Our methods are most appropriate for research-designed concept tests with distractors based on common wrong answers, but they may also prove suitable for examining multiple-choice assessments developed using other research-based approaches (such as think-aloud protocols). Longitudinal studies using such instruments may yield insights about how student reasoning manifests and develops over time *in situ*, providing clues about how various mental models emerge (Brown, 2014) and ultimately about how people transition from novices to experts (Burkholder et al., 2020).

We base our approach on a flexible multidimensional IRT model for multiple-choice data known as the Multidimensional Nominal Categories Model (MNCM, discussed in more detail in Section 3). In contrast with unidimensional IRT—which ranks testees on a single monotonic scale corresponding to ability or some other psychological trait of interest—multidimensional models such as the MNCM rank testees along *several* distinct dimensions, each capturing different aspects of the interaction between the latent mindsets of the testees and the constructs of the test. A combination of parameter constraints and standard factor-analytic rotation methods then aids in finding a representation of these dimensions that allows insightful interpretation. This approach is, in essence, a form of item factor analysis (Bock et al., 1988), though it is perhaps better understood as “category factor analysis” given the flexibility of the MNCM to capture within-category multidimensionality.

While our methodological choices were made with an eye to identifying student misconceptions from concept-test data, we note that the method itself is agnostic to the meaning of any traits it discovers. Some of these may indeed be misconceptions—and we have seen preliminary evidence that many are—but others might represent misunderstandings of the questions themselves or even factors outside the intended scope of the test (such as graphical literacy on a test of Newtonian physics). Ultimately, any discovered traits will require interpretation and eventually validation, and in this sense we view the method as exploratory and complementary to other modes of research (on misconceptions or otherwise).

This paper begins with a brief introduction to a classic IRT model for multiple-choice questions—the Nominal Categories Model (Bock, 1972)—followed by the MNCM which can be understood as one of its most general multidimensional extensions. We then present a Bayesian implementation of the MNCM based on a variational inference approach with hierarchical priors, which we find to be robust to small sample sizes while not requiring careful tuning of item prior widths to match the dataset. Using simulated data, we validate this implementation in its unidimensional limit against existing open-source software implementing the marginal maximum likelihood method for nominal responses. We also study how the number and quality of recoverable dimensions depends on the sample size and the strengths of the item-testee interactions in each dimension, and discuss an emergent dimensionality self-selection property of the method.

We then present some preliminary results from the application of this procedure to ~10,000 students' responses to the Force Concept Inventory (FCI), the original research-designed multiple-choice concept test in STEM (Hestenes et al., 1992). The Bayesian MNCM method extracts 14 dimensions from these data, and we choose an exploratory bi-factor rotation method (which promotes sparse loadings in all but one dimension) to yield interpretable results. In addition to identifying a prominent general dimension highly correlated with the raw test score, we find that some of the sparse dimensions are identifiable misconceptions familiar from the literature on student misconceptions in introductory Newtonian mechanics. Two illustrative examples are discussed in this work, with a more comprehensive analysis reserved for a forthcoming paper (in preparation). While additional research is needed to establish the broader validity of these findings, they nevertheless serve as a promising indicator of the value of our method.

We conclude by stressing the usefulness of methods such as ours for both formative assessment and research. Although the techniques of our approach are solidly within the purview of IRT, our application of these to study details of not-knowing within a domain is relatively novel in a discipline traditionally devoted to measuring or certifying knowing within a domain. Future iterations of these methods could provide guidance to teachers (by informing them of the particularly severe misconceptions of their students) or help researchers design and improve other assessments, especially where student misconceptions are not so well studied as in mechanics. Our work invites further exploration of the similarities of misconceptions across different universities or skill levels of the students and application to pre- and post-test data to reveal the effects of instruction and the changes in student thinking it might catalyze.

## 2 The nominal categories model

On a multiple-choice test, each question (“item” in IRT parlance) contains a limited set of possible response alternatives (“categories”), of which testees may choose only one. In some cases, these categories have an inherent ordering; an assessment of anxiety symptoms may use categories ranging from “never describes me” to “always describes me,” with various gradations in between. However, many multiple-choice tests contain items in which categories are qualitative with no inherent ordering, or in which an ordering may not be known *a priori*. Responses to such items only encode *which* category was selected—without any associated ranking or quantitative value—and are referred to as nominal (in contrast to ordinal) responses.

While nominal multiple-choice questions are sometimes graded as simply “correct” or “incorrect” in an educational context, doing so discards information about the items and testees conveyed by the specific distractors selected. This is true even in a unidimensional case where our concern is ranking students along a single ability scale: even here, some distractors may be “more wrong” than others.

An attractive alternative to dichotomous grading is to use a model specifically intended for nominal data. Perhaps the best known of these is the Nominal Categories Model (NCM; Bock, 1972), also often called the Nominal Response Model in IRT literature and software. The NCM assigns each student a “response tendency” for each category in an item, with the probability of selecting a particular response category related to these by the multinomial logistic function (the exponent of the tendency divided by the sum of the exponents of the tendencies of all categories in the item). Mathematically, the probability that student *s* will select category *c* as their response $r_{s}^{\left(i\right)}$ to item *i* is


(1) $$\begin{array}{l} p\left(r_{s}^{\left(i\right)}=c|t_{s}^{\left(i\right)}\right)=\frac{\exp t_{s}^{\left(i,c\right)}}{\overset{C}{\underset{c^{\prime }=1}{\sum }}\exp t_{s}^{\left(i,c^{\prime }\right)}}, \end{array}$$

where $t_{s}^{\left(i\right)}=\left[t_{s}^{\left(i,1\right)},t_{s}^{\left(i,2\right)},\ldots ,t_{s}^{\left(i,C\right)}\right]$ is a vector of the aforementioned response tendencies for the student and item and *C* is the number of categories. For the NCM, these tendencies are given by a linear functions of some latent student ability *θ*_*s*_,


(2) $$\begin{array}{l} t_{s}^{\left(i,c\right)}=\theta_{s}a^{\left(i,c\right)}+b^{\left(i,c\right)}, \end{array}$$

where *a*^(*i,c*)^ is a slope parameter for category *c* of item *i*, and *b*^(*i,c*)^ is an intercept parameter for the same.^1^

In addition to being substantially more flexible than a dichotomous model, the NCM also has a plausible psychological interpretation which makes it well suited to our use case: it may be understood as approximating a comparative choice process in which each student assigns some (unobserved) preferences to all response categories in an item, then chooses the category for which their preference is greatest.^2^ An excellent exposition of this topic is presented by Thissen et al. (2010, p. 49–50 & 66–70). This understanding forms the basis for the theoretical explainability of the model parameters in Equation 2.

The latent ability parameter *θ*_*s*_ has a straightforward meaning which matches that of dichotomous IRT: it measures a student's overall skill level in the test domain relative to that of other students. The slope parameters, *a*^(*i,c*)^, require more care to explain due to the non-linear nature of Equation 1, especially since the tendencies for *all* categories in an item are present in the denominator of the response probability (a form of normalization which is necessary to ensure that the probabilities sum to one across all possible responses). Despite the apparent similarity to a dichotomous two-parameter logistic (2PL) model in slope-intercept form, the coupling between category probabilities introduced by normalization means that the *a*^(*i,c*)^ terms in the NCM cannot be thought of as discriminations. Rather, they provide a relative measure of the association between latent ability and each of the response categories, and as such serve to indicate the empirical ordering of the categories in an item.

The intercept parameters, *b*^(*i,c*)^, may be understood as a measure of how inherently attractive each category is to a student with ability *θ*_*s*_ = 0; this corresponds to an “average student” under the typical (albeit arbitrary) IRT convention of fixing the population mean of the abilities to zero. Even if this convention is assumed, though, we must stress that ‘attractiveness to the average student' is *not* the same as ‘average attractiveness to students,' and that these two are not even guaranteed to be monotonically related. Consequently, we view these terms as somewhat less suited to direct interpretation compared to *θ*_*s*_ and *a*^(*i,c*)^.

Nevertheless, the intercept parameters contribute essential flexibility to the model. For items with three or more categories, the combination of the normalization step in Equation 1 and the per-category intercepts in Equation 2 permits the NCM to model category response curves with intermediate maxima—that is, those having a response probability which peaks at some finite *θ* and decays to zero in both limits as *θ* → ±∞. Such curves frequently occur in real test data (Pérez-Lemonche et al., 2019; Stewart et al., 2021), and are produced by the NCM whenever *a*^(*i,c*)^ for some category *c* lies between *a*^(*i*,*c*^′) and *a*^(*i*,*c*^″), where *c*′ and *c*″ are other categories in the same item. This is clearly not possible with dichotomous models such as the 2PL, in which all incorrect responses are lumped together and have a response probability which decreases monotonically with increasing ability.

## 3 The multidimensional nominal categories model

Since different distractors on research-validated instruments typically reflect different misconceptions, and since we want to interpret the different dimensions of the latent ability space in correspondence with different (mis)conceptions, we need a multidimensional model where different dimensions indicate different sets of wrong responses. Such an extension of IRT to *D* dimensions would allow each response category in an item to have a unique slope and intercept term for each dimension, thus allowing categories to have distinct directions in *D*-dimensional space. The tendencies in such a model would then become:


(3) $$\begin{array}{l} t_{s}^{\left(i,c\right)}=\overset{D}{\underset{d=1}{\sum }}\left[a_{d}^{\left(i,c\right)}\theta_{s}^{\left(d\right)}+b_{d}^{\left(i,c\right)}\right]. \end{array}$$

Having unique intercepts for each dimension is redundant however as these combine into a single constant in the tendency expression. Denoting this as *b*^(*i,c*)^ (with no subscript) yields the typical form of the Multidimensional Nominal Categories Model (MNCM) first introduced by Takane and de Leeuw (1987) in which the tendency for student *s* to give response *c* to question *i* is


(4) $$\begin{array}{l} t_{s}^{\left(i,c\right)}=\overset{D}{\underset{d=1}{\sum }}\theta_{s}^{\left(d\right)}a_{d}^{\left(i,c\right)}+b^{\left(i,c\right)}. \end{array}$$

Alternative parameterizations of the MNCM have also been proposed in more recent literature, most notably by Thissen et al. (2010). In place of the slope parameters, they use a product of a *D*-dimensional “overall discrimination” vector (which is shared across all categories in an item) with a set of *C* scalar “scoring function values” (shared across all dimensions) which dictate the relative ordering of the categories. Subsequent presentations also permit these scoring function values to be multidimensional (Thissen and Cai, 2016). Such parameterizations have the advantage of providing an intuitive measure of overall item discrimination and direction, much like a multidimensional 2PL model. As our approach to identifying misconceptions relies on examining the relationships between individual distractors and each latent ability dimension, we will use the more traditional parameterization in Equation 4 which provides more directly interpretable parameters for this application.

In summary, the MNCM has several important properties that make it well suited to the task of misconception analysis:

- It has a plausible psychological basis, providing a theoretical foundation for its use in understanding student though processes.
- It is designed for nominal multiple-choice data. No inherent ordering is imposed on the response categories *a-priori*, and the predicted probabilities across possible responses to an item always sum to one, reflecting the constraint that students can select only one of the available choices.
- It is multidimensional at the level of individual categories. Each distractor can have its own direction (vector) in the multi-dimensional ability space, such that different distractors in the same item may reflect different misconceptions.

## 4 The MNCM-Bayes method

Fitting the MNCM to real data is challenging due to the large number of free model parameters and the small fraction of response patterns that are ever observed (e.g., a 30-item multiple choice test like the FCI with five categories per item has approximately 10^21^ distinct ways in which the questions can be answered). This can lead to large errors when using maximum-likelihood-based fitting methods as some parameters may have little data informing their estimates, especially with smaller sample sizes.

In order to better recover model parameters at small sample sizes, we take a Bayesian approach to fitting the MNCM as suggested by Revuelta and Ximénez (2017). This section describes the details of our resulting method, which we will refer to in this paper as *MNCM-Bayes*, as well as providing relevant background about various elements of our approach. We will begin with a discussion of the invariances present in the MNCM and our procedure for imposing identification constraints; while this is the final step in our method procedurally, it is helpful to introduce it first as it aids in understanding some aspects of the earlier steps.

### 4.1 A note on notation

Throughout this section we will make use of matrices (i.e., two dimensional arrays of parameters) to simplify our explanations. We will denote such matrices by bold, italicized capital letters—for example, ***A*** for a matrix containing all slope parameters—and denote their individual elements by italicized lower case letters as we have done thus far, with row indices indicated by subscripts and column indices indicated by parenthesized superscripts. Bold lower case letters will indicate row or column vectors.

In the case of matrices with dimensions of size *IC* (corresponding to the set of all response categories on all items), we use the index pair (*i,c*) as a shorthand for the index corresponding to category *c* of item *i*. The definitions and shapes of several key matrices are provided in Table 1 for reference.

**Table 1:** Symbols and dimensions for key matrix-valued quantities.

**Symbol**	**Description**	**Dimensions**
***R***	Responses (observed)	*S*×*I*
***Θ***	Student abilities/latent traits	*S*×*D*
***A***	Category slopes	*D*×*IC*
***b***	Category intercepts	1 × *IC*

### 4.2 Identifying the MNCM

Specifying a procedure to uniquely identify the MNCM is required because the model has a number of symmetries that result in invariances—mathematical transformations to the model parameters that leave the predicted probabilities unchanged. Consequently, varying a set of free parameters to find the best fit to a data set does not uniquely determine those parameters, which are arbitrary within variations that honor the invariances. In order to compare results from different data sets—or even from different subsets of the same data—we must impose additional constraints on the parameters in order to *identify* (uniquely specify) the model. A familiar example from dichotomous 2PL IRT is that shifting item difficulties and student abilities by the same amount does not change the predicted probability for student *s* to answer item *i* correctly; typically, the corresponding identification constraint for this invariance is to set the mean ability to zero.

The greater mathematical complexity of the MNCM results in a greater number of symmetries, and hence a larger number of identification constraints are necessary to specify reproducible results. Additionally, for a multidimensional model in which the probabilities depend on a scalar product (like the MNCM; see Equation 4), rotations of the vector space leave the probabilities unchanged; we exploit several affordances of this when fitting data (both synthetic data and actual student data).

Our identification procedure below is a specification of certain constraints on model parameters (or on properties of sets of parameters such as means or standard deviations), and the parameter transformations we use to effect these constraints. Since by definition the identification procedures do not change the probabilities (and often do not even change the tendencies), they can be applied either while fitting the model to data as an integral part of that process, or afterward as a separate post-processing step on the estimated parameters.

Note that in order to leave the predicted probabilities unchanged, enforcing identification constraints on some parameters often requires making corresponding changes to other parameters linked by the model invariances.

#### 4.2.1 Identification procedure

We center ***Θ*** at zero over the sample (standard practice in IRT) for every dimension by subtracting the mean ability ${\langle \theta^{\left(d\right)}\rangle }_{s}$ from each $\theta_{s}^{\left(d\right)}$; in order to keep the tendencies (Equation 4) and thus also the probabilities (Equation 1) unchanged, this requires that we also shift each *b*^(*i,c*)^ by an amount


(5) $$\begin{array}{l} \text{Δ}b^{\left(i,c\right)}=\overset{D}{\underset{d=1}{\sum }}a_{d}^{\left(i,c\right)}{\langle \theta^{\left(d\right)}\rangle }_{s}. \end{array}$$

We then shift the item parameters ***A*** and ***b*** in one of two ways depending on the nature of the data being analyzed. For data derived from multiple-choice assessments with a “correct answer” category, we set both $a_{d}^{\left(i,\text{correct}\right)}$ and *b*^(*i*, correct)^ to zero for each item (by means of a suitable opposite shift of all *a*^(*i,c*)^ and *b*^(*i,c*)^); identifying in this way highlights the distractors that are most different from the correct answer, which is desirable for misconception analysis. When no obvious reference category exists (such as in our synthetic data studies), we set the means ${\langle a_{d}^{\left(i,c\right)}\rangle }_{c}$ and ${\langle b^{\left(i,c\right)}\rangle }_{c}$ to zero instead. These two approaches are sometimes known as *simple constraints* and *deviation constraints*, respectively (see Revuelta and Ximénez, 2017, p. 2). In either case, these changes shift the tendencies (Equation 4) by the same amount across all categories in an item, which multiplies all terms in Equation 1 by the same factor and hence leaves the probabilities unchanged.

Additional invariances occur because the tendencies given by Equation 4 contain a scalar product of ***A*** and ***Θ***, the slope matrix and the student abilities respectively. In consequence, ***A*** and ***Θ*** can be transformed by any invertible linear mapping: this includes any combination of scaling (e.g., increasing ***a***_*d*_ and decreasing ***θ***_*d*_), rotation, shearing, reflection (sign reversal of both ***a***_*d*_ and ***θ***_*d*_), or permutation of dimensions.

The scaling invariance allows us to constrain the variance of the ***Θ***-distribution to be unity in each dimension, as frequently done in IRT. We initially identify rotation and shearing by specifying that ***Θ*** and ***A*** have diagonal covariance matrices—the data therefore determines the scale of the estimated ***a***_*d*_ vectors, which we rank in order of decreasing variance by default to identify permutation. These last identification constraints are often short-lived, though, as when analyzing real data it is common to further transform model parameters to maximize interpretability (e.g., by applying factor-analytic rotation methods as we discuss in Section 6.1).

### 4.3 Bayesian modeling of the MNCM

As mentioned earlier, we take a Bayesian approach to fitting the MNCM. In the Bayesian paradigm, the probability of a student selecting a particular response category is still modeled by Equations 1, 4; however, every model parameter [each individual $a_{d}^{\left(i,c\right)}$, *b*^(*i,c*)^, and $\theta_{s}^{\left(d\right)}$] is treated as having an entire probability *distribution* over possible values rather than a single “optimal” value. This provides a principled way of modeling the effects of parameter uncertainty. In addition, Bayesian methods allow us to incorporate reasonable prior beliefs about how parameters will be distributed. For example, it is typical to assume *a priori* that population values will be more-or-less normally distributed. These two qualities make Bayesian modeling well suited to the challenges of fitting the real data described earlier.

Our choice of prior probability distribution is based on the work of Natesan et al. (2016), who recommended the use of hierarchical models for Bayesian IRT:


(6) $$\begin{array}{l} \begin{array}{c} \alpha_{d},\beta \sim \text{HalfCauchy}\left(5\right)\\ a_{d}^{\left(i,c\right)}\sim \text{Normal}\left(0,\alpha_{d}\right)\\ b^{\left(i,c\right)}\sim \text{Normal}\left(0,\beta \right)\\ \theta_{s}^{\left(d\right)}\sim \text{Normal}\left(0,1\right) \end{array} \end{array}$$

In a hierarchical model, some of the prior probability distributions are not fully specified but are instead parameterized by additional “higher level” random variables [here, α_*d*_ and β, which serve as scale parameters for the priors of $a_{d}^{\left(i,c\right)}$ and *b*^(*i,c*)^ respectively]. These variables are then learned from the data. Such an approach allows item parameters with high-confidence estimates to inform the scale of the priors, which in turn better stabilizes estimates for the parameters which have less supporting data.

Note that all prior means for both ability and item parameters are fixed at zero. Due to the invariances of the model, this does not result in any loss of generality. Instead, it serves as a “soft” identification constraint which stabilizes the location of the parameters during the fitting process. Similarly, the scale of ***Θ*** (and therefore of ***A***) is identified by the fixed variance of the $\theta_{s}^{\left(d\right)}$ prior, and the rotation and shearing of the model are partly identified by the use of independent priors for each dimension (which implicitly results in diagonal covariance matrices for both ***A*** and ***Θ***, again without any loss of generality). The signs and ordering of dimensions are left unidentified until after the fitting process, though this does not seem to adversely affect convergence due to our choice of fitting method discussed below.

### 4.4 Approximate Bayesian inference

The process of fitting a Bayesian model to data is called *inference*, and its output is an updated joint probability distribution over all model parameters called the *posterior*. Given a set of observed responses ***R***, a prior probability distribution p(Ƶ) over parameters Ƶ≡{Θ,A,b,α,β}, and a response model p(R∣Ƶ) describing the probabilities of the observations given the parameters (Equations 1, 3 for the MNCM), then the posterior p(Ƶ∣R) may be found by applying Bayes' rule:


(7) $$\begin{array}{l} p\left(Ƶ|R\right)=\frac{p\left(R|Ƶ\right)p\left(Ƶ\right)}{p\left(R\right)} \end{array}$$

This equation, however, is deceptive in its simplicity; except in special cases, it is not possible to find a closed-form analytical expression for the posterior, and it must be approximated numerically.

Our work uses a variational inference (VI) approach in order to approximately solve Equation 7. VI methods work by re-framing Bayesian inference as an optimization problem: given an approximate (but analytically tractable) parameterized probability distribution, a numerical optimizer searches for the parameters which bring this approximant closest to the true posterior. Surprisingly, it is possible to do this without ever computing the true posterior by instead maximizing a surrogate objective function known as the Evidence Lower Bound (ELBO); further details are widely available in the academic literature (e.g. Blei et al., 2017). In addition to being considerably faster than the more typical Markov Chain Monte Carlo methods used for Bayesian inference, this optimization-based approach allows VI to converge to just one of the many (arbitrary) permutation of dimensions and signs in an under-identified model.

While the form of the approximate posterior distribution in VI may be arbitrarily complex, a popular and quite effective simplification is to treat each random variable in the model as having its own independent univariate posterior distribution. This is known as the mean-field approximation. For all real-valued model variables ($\theta_{s}^{\left(d\right)}$, $a_{d}^{\left(i,c\right)}$, and *b*^(*i,c*)^), we choose as the approximate posterior a normal distribution parameterized by a mean and a standard deviation, both of which may be freely varied by the optimizer.

The approximate posterior for the higher-level random variables α_*d*_ and β require additional care since they cannot take negative values. This constraint is handled by introducing surrogate real-valued variables—with posteriors modeled by unconstrained normal distributions as above—and mapping these to positive numbers using an appropriate bijection (e.g., the softplus function *x* ↦ log[1 + exp(*x*)]).

### 4.5 Dimensionality assessment

When selecting the dimensionality of a multidimensional IRT model, it is important to balance improvements in predictive ability from additional dimensions against the added degrees of freedom thereby introduced. Not doing so inevitably leads one to select a model which performs well only on the specific data used to estimate its parameters, but fails to explain new data generated from the same underlying statistic process or yields psychologically meaningless parameter estimates. This problem is known as *overfactoring*—a type of overfitting—and is often dealt with in a classical maximum-likelihood context by using likelihood-ratio tests or information criteria to compare models with different dimensionalities (van der Linden, 2016, chs. 17 & 18).

Bayesian methods are not entirely immune to overfactoring, though their principled inclusion of parameter uncertainty does provide some built-in protection against it. Several approaches to Bayesian dimensionality assessment were recently compared by Revuelta and Ximénez (2017) for non-hierarchical MNCM models with up to three dimensions estimated using Markov Chain Monte Carlo methods. The authors recommended the use of a standardized generalized dimensionality discrepancy measure (SGDDM) in this context, noting that alternatives based on discrepancy measures struggled to correctly identifying the dimensionality of synthetic data.

In our work, we find that using a hierarchical model in combination with variational inference performs simultaneous parameter estimation and dimensionality selection: even when a *D*-dimensional model is specified, some of those may be “turned off” during inference (by setting the corresponding α_*d*_ and ***a***_*d*_ to ~0) when the observed data provide insufficient evidence to confidently estimate their slopes. This results in considerable robustness to overfactoring as illustrated by our simulation study in Section 5.

### 4.6 Implementation details

The method described above was implemented in the Python programming language (version 3.11). We leveraged the NumPyro probabilistic programming framework (Phan et al., 2019) to define the probabilistic model, automatically generate a corresponding mean-field normal approximating posterior (including surrogate variables and bijections for α_*d*_ and β), and perform approximate inference using NumPyro's built-in Stochastic Variational Inference (SVI) algorithm.

As the name might suggest, SVI relies in part on random sampling to evaluate and optimize the variational objective function, and must therefore be paired with a noise-tolerant optimization routine. We use the Adam optimizer (Kingma and Ba, 2014) configured with a variable “learning rate” parameter which is programmed to decrease from 0.05 to zero on a predetermined 30,000-step schedule.^3^

While SVI will often converge even if the posterior means of all variables are randomly initialized, we attempt to provide a more reasonable starting point for optimization by computing initial guesses of these using a fast IRT approximation (Zhang et al., 2020),^4^ resorting to random initialization only when this approach does not yield a solution. The posterior standard deviations of all variables are initialized to a fixed value of 0.1, which is the default value for mean-field normal posteriors in NumPyro.

The raw, post-optimization outputs of the SVI algorithm consist of an estimated mean and standard deviation for each variable in the approximate mean-field posterior—that is, for each individual $a_{d}^{\left(i,c\right)}$, *b*^(*i,c*)^, $\theta_{s}^{\left(d\right)}$, α_*d*_, and β. However, these raw outputs are only weakly identified by the Bayesian prior during inference, and require post-processing to exactly impose the identification constraints described in Section 4.2. We perform this step analytically, using the posterior standard deviations to account for uncertainty when finding the covariance matrices of ***A*** and ***Θ*** (which are needed to fully identify the scale and rotation of the model) and the variance of ***b*** (which is included for completeness). The standard deviations are not used further in this work; we examine only the means of the identified parameters, which correspond to an *expected a posteriori* (EAP) solution.

Lastly, any dimensions inactivated during inference (Section 4.5) are omitted from the final estimates. These dimensions may be easily detected by computing the magnitudes of the estimated slope vectors and applying a simple threshold criterion; we use ||***a***_*d*_|| ≤ 0.005.

The final identified outputs of our method therefore include:

- $\hat{D}$, the total number of retained dimensions (with $\hat{D}\leq D$);
- $\hat{A}$, the matrix of EAP category slope estimates;
- $\hat{b}$, the vector of EAP category intercept estimates;
- $\hat{\Theta }$, the matrix of EAP ability estimates;
- $\hat{\text{Cov}\left[A\right]}$, the estimated $\hat{D}\times \hat{D}$ posterior covariance matrix of all the slopes; and
- $\hat{\text{Var}\left[b\right]}$, the estimated posterior variance of all the intercepts.

## 5 Tests with synthetic data

In this section we apply the MNCM-Bayes method to synthetic response data (generated using the MNCM as the true underlying model) and study its ability to recover the multidimensional slope parameters used in the synthesis. We explore how this parameter recovery performance varies across a range of sample sizes and ***a***_*d*_-vector scales, and also compare the method's results to those obtained with established IRT software in a limiting unidimensional case.

### 5.1 The synthesized data

Our synthesized datasets each comprised a set of synthetic student and item parameters ***Θ***, ***A***, and ***b*** (all identified according to the constraints in Section 4.2) and a corresponding synthetic response matrix ***R***. All datasets used *I* = 30 items and *C* = 5 categories per item, matching the structure of the real data we will analyze in Section 6. The standard deviation of the ***b*** vector was fixed at 1.5, which is also consistent with that found later for the real dataset; this yielded synthetic data with a range of observed responses fractions across the categories in each item.

For the multidimensional simulation study, datasets were generated with *D* = 9 synthesized dimensions. Six sample size levels *S* ∈ {50, 100, 200, 600, 2, 000, 10, 000} and two ***A***-matrix covariance structures (described below) were explored according to a fully-crossed design. For each condition, 100 replications were generated with different pseudo-random parameter values for each replication, yielding a total of 1,200 datasets.

The invariances of the MNCM afford us substantial flexibility in choosing a covariance structure for the synthesized ***A*** matrices. Of note, *all* possible covariance matrices for ***Θ*** and ***A***—even those with arbitrary structure and multiple highly-correlated dimensions—are expressible as diagonal matrices in *some* choice of reference frame due to these invariances,^5^ which allows us to synthesize ***A*** to have uncorrelated dimensions without any loss of generality. The standard deviations of these dimensions were selected to span a fairly wide gamut in order to explore the limits of parameter recovery using our method; we fix the standard deviation of the first dimension to 1.0 and specify that each subsequent dimension is smaller than the previous one by a factor γ ∈ {0.8, 0.512}:


(8) $$\begin{array}{l} StdDev\left[a_{d}\right]=1.0\times \gamma^{\left(d-1\right)}. \end{array}$$

The first condition, γ = 0.8, yields data in which the smallest dimension accounts for approximately 1% of the overall variance in the synthesized tendencies. The second condition yields data with a much more rapid decrease in variance (as might be expected in a real dataset with fewer significant factors), with the specific choice of γ = 0.8^3^ = 0.512 intended to facilitate comparison across the two conditions: the standard deviations of dimensions 1–3 in the γ = 0.512 data exactly match those of dimensions 1, 4, and 7 in the γ = 0.8 data.

For the unidimensional simulation study comparing the Bayesian method to an established software package implementing Marginal Maximum Likelihood Estimation (MMLE), datasets were generated with *D* = 1, sample sizes *S* ∈ {50, 100, 200, 600, 2, 000, 10, 000}, and a standard deviation of 1.0 for the sole slope vector ***a***_1_. Again, 100 replications were generated per condition, yielding an additional 600 datasets for this second study.

#### 5.1.1 Student parameter synthesis

The ***Θ*** matrices were synthesized by drawing samples from a *D*-dimensional standard normal distribution and then standardizing this set of samples to have zero mean in all dimensions and an identity covariance matrix. This added standardization step (and those applied to the item parameters below) served two goals: imposing exact identification constraints to facilitate later comparison with the recovered parameters, and reducing the sample-to-sample variation in the scale of the model due to random sampling variation at smaller sample sizes.

Within each replication, a “nested” structure was used for the student parameters, such that the matrix for each sample size included those of all smaller sample sizes as subsets. That is, ***Θ***|_*S* = 100_ consisted of ***Θ***|_*S* = 50_ concatenated with an additional (and separately standardized) 50 rows, ***Θ***|_*S* = 200_ included ***Θ***|_*S* = 100_ plus 100 new rows, and so on. This was done both to reduce variance across conditions and to permit direct comparisons between parameter estimates if desired.

#### 5.1.2 Item parameter synthesis

The ***A*** matrices and ***b*** vectors were generated using a similar procedure to ***Θ***. In order to allow the covariance between slopes and attractivenesses to be exactly specified, these two variables were initially treated as a single column-wise concatenated matrix [***A***^⊺^|***b***^⊺^] having dimensions *IC* × (*D* + 1), with rows drawn from a (*D* + 1)-dimensional standard normal distribution. As in Section 4.2, the combined parameters were identified such that each group of rows corresponding to a given item had zero mean in all *D* + 1 columns. The entire matrix was then standardized to have an identity covariance matrix, scaled to achieve the desired standard deviations for each column, and finally split and transposed into the individual variables ***A*** and ***b***.

#### 5.1.3 Response synthesis

The response matrices ***R*** were generated by sampling each element from a categorical distribution with probabilities given by Equations 1, 4. As with the student parameters, a nested sampling approach was used, such that the response matrix for each sample size in a given replication included as subsets the response matrices of all smaller sample sizes.

### 5.2 Aligning recovered and synthesized parameters

Our interest in finding misconceptions means that, when studying real data, we will need to rotate the coordinate system of our results to associate each dimension with an interpretable concept (common practice in exploratory analysis methods; see Section 6.1). We are therefore interested in evaluating how well the method recovers the *basis-independent* information present in our synthesized parameters and especially in the ***A*** matrix, rather than the particular coordinate system in which it initially extracts this information (which is arbitrary and does not affect the response probabilities due to the rotational invariance property of the MNCM). We achieving this by aligning the coordinate systems of the synthesized and recovered parameters prior to computing any evaluation metrics.

Note that using the same identification criteria for both sets of parameters does somewhat succeed in aligning their coordinate systems. Even so, the identified coordinate directions may themselves be sensitive to small errors in the parameters; this procedure may therefore lead to inflated errors which are due more to (arbitrary) differences in rotation than to differences which actually affect the response probabilities.

In this work, we use an orthogonal Procrustes procedure (Schönemann, 1966) to align the recovered and synthesized parameters while respecting the invariances of the model. We first find an orthogonal matrix ***Q*** which best maps ***Θ*** to $\hat{\Theta }$ in the least-squares sense, i.e., which minimizes


(9) $$\begin{array}{l} ||\Theta Q-\hat{\Theta }{||}_{F}^{2}\;\text{s.t. }QQ^{⊺}=I \end{array}$$

where $||\cdot {||}_{F}^{2}$ is the squared Frobenius norm (equal to the sum of squares of all matrix elements). This matrix is then used to rotate the synthesized parameters to allow direct comparisons with the estimates:


(10) $$\begin{array}{l} \begin{array}{c} \Theta^{⋆}=\Theta Q\\ A^{⋆}=Q^{\text{⊺}}A \end{array} \end{array}$$

Note that such rotations do not alter the predicted probabilities in Equations 1, 4.

The choice to align parameters to the reference frame of the estimates—as opposed to that of the synthesized parameters—is a deliberate one. Because of the self-limiting dimensionality of the MNCM-Bayes method, the number of dimensions extracted may be smaller than the number of dimensions synthesized. Remaining in the reference frame of the estimates allows us to limit our analysis to only these $\hat{D}\leq D$ extracted dimensions, and also allows us to examine parameter recovery metrics for each extracted dimension individually. In the reference frame of the synthesized parameters however, there is no longer a one-to-one correspondence between these extracted dimensions and individual coordinate directions, and limiting the analysis to the $\hat{D}$-dimensional subspace of extracted dimensions becomes difficult or impossible.

### 5.3 Results and discussion

For this paper, we focus exclusively on the recovery of the slope parameters $a_{d}^{\left(i,c\right)}$ as these provide the most information about the structure and content of an assessment instrument (which is our current research focus). We leave exploration of ***Θ*** and ***b*** for future work.

We evaluate parameter recovery separately for each dimension. Our metric of choice is the squared Pearson correlation coefficient—also known as the coefficient of determination—computed between the synthesized, Procrustes-aligned $a_{d}^{⋆\left(i,c\right)}$ values and their estimated counterparts ${\hat{a}}_{d}^{\left(i,c\right)}$:


(11) $$r^{2}\text{​}\left[a_{d}^{⋆},{\hat{a}}_{d}\right]\equiv \frac{{\left[\sum_{i,c}\left(a_{d}^{⋆(i,c)}\text{​}-{\langle a_{d}^{⋆}\rangle }_{i,c}\right)\text{​}\left({\hat{a}}_{d}^{\left(i,c\right)}\text{​}-{\langle {\hat{a}}_{d}\rangle }_{i,c}\right)\right]}^{2}}{{\sum_{i,c}\left(a_{d}^{⋆(i,c)}\text{​}-{\langle a_{d}^{⋆}\rangle }_{i,c}\right)}^{2}{\sum_{i,c}\left({\hat{a}}_{d}^{\left(i,c\right)}\text{​}-{\langle {\hat{a}}_{d}\rangle }_{i,c}\right)}^{2}}.$$

We intentionally use a correlation-based metric instead of the more common root mean squared error (RMSE) in order to forgive differences in overall scale, which do not affect the subjective interpretation of the recovered parameters.

The *r*^2^ metric ranges from 0 to 1 and may be understood as the fraction of variation in the slope estimates attributable to variation in the (rotated) ground-truth values. This definition implies that values of *r*^2^ are analogous to reliability coefficients, except that these apply to recovered slope rather than test scores. We therefore suggest similar norms be used when evaluating *r*^2^: values greater than 0.9 suggest sufficient accuracy for interpreting individual slopes, while those as low as 0.7 may still provide value when interpreting multiple slopes in aggregate.

#### 5.3.1 Multidimensional slope recovery

**Figure 1:**
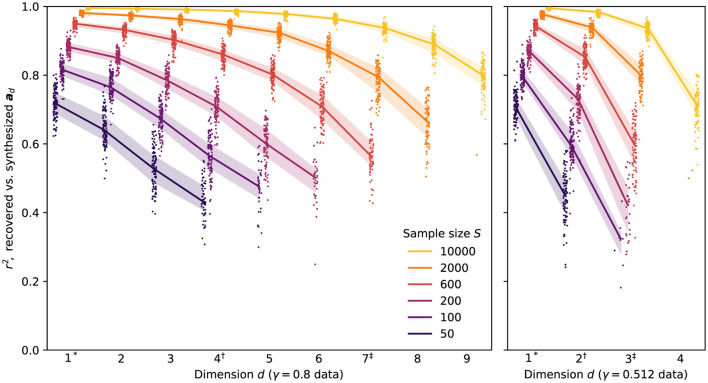
Per-dimension coefficients of determination for recovered vs. synthesized slopes, after Procrustes alignment, for all multidimensional synthesized datasets. Points show results from individual replications, with jitter and horizontal offsetting used to reduce overplotting. Solid lines and shaded bands show median and interquartile ranges, respectively, whenever five or more replications are present for a given γ, *S*, and *d*. To facilitate comparison between the two γ conditions, the superscripts *, † and ‡ are used to indicate dimensions with equal standard deviations.

The *r*^2^ coefficients for the recovered slopes in the nine-dimensional simulation study are summarized in Figure 1. Recovery ranged from excellent (*r*^2^>0.9 for most dimensions at *S* = 10, 000) to poor (only marginally-acceptable performance in the first dimension for *S* = 50, with questionable results beyond this), though this was largely expected given the span of sample sizes tested.

Perhaps the most conspicuous feature of the results is the self-limiting dimensionality of the MNCM-Bayes method. At the largest sample size tested (*S* = 10, 000), the method was able to recover coefficients of all synthesized dimensions in the γ = 0.8 data across the majority of experimental replications. As sample size decreases, however, the effects of smaller dimensions become increasingly difficult to distinguish from those of random noise in the data, increasing the risk that extracted parameters will differ from those of the true underlying model. Rather than yielding meaningless results for these dimensions, we find that method settles on solutions in which only a subset of the *D* = 9 estimated dimensions are used, with the remainder having EAP estimates set close to zero—effectively pruning them from the final model.^6^ This behavior provides considerable robustness to both overfactoring and overfitting: in general, the method appears to include dimensions only when it is confident that the resulting estimates contain real information about the underlying slopes, not simply when doing so would increase the likelihood of the observed responses on a particular dataset.

At the same time, we recognize that merely containing information is too low a bar when it comes to meaningful interpretation of model parameters. In this sense, our results underscore the critical role that large sample sizes play in allowing us to draw conclusions about the slope coefficients in lower-variance dimensions, especially if we wish to interpret these individually rather than in aggregate.

Comparing the results across the two levels of γ, we find that recovery performance is quite similar for dimensions with similar overall standard deviations (indicated by matching superscripts on the dimension numbers in the two subplots). For example, the results for *S* = 2, 000 show similar performance (*r*^2^≈0.8) in dimension 7 of the γ = 0.8 data and dimension 3 of the γ = 0.512 data—both of which were synthesized with standard deviations of 0.262. This is true despite there being a greater *number* of dimensions having at least this standard deviation in the γ = 0.8 data, suggesting that the dimensionality of the data plays a relatively minor role in determining parameter-recovery accuracy in any given dimension compared to the scale of the underlying ***a***_*d*_ vector (and of course the sample size).

The number of dimensions retained by the method also seems to depend primarily on the scales of the ***a***_*d*_ vectors at any given sample size. At *S* = 600 for example, the method yielded models with up to seven dimensions in the γ = 0.8 data and only three dimensions in the γ = 0.512 data, but in either case the smallest recovered dimension had StdDev[***a***_*d*_]≈0.26. This result also implies that sample size should not be seen as limiting the *number* of dimensions that can be recovered by the method, but rather the *smallest* dimension that can be reliably extracted given the limited information about the model parameters provided by each response.

#### 5.3.2 Agreement with established IRT software

We also compared the slope recovery of our method to those obtained with the widely adopted “mirt” package for the R language (Chalmers, 2012; R Core Team, 2021) using default settings, which corresponded to MMLE with a standard-normal ability prior and no item parameter priors. This comparison was limited to a unidimensional model since, as of the date of submission, “mirt” only supports the more restricted (Thissen et al., 2010) form of the MNCM in which all categories slopes for a given item are assumed to share the same direction in the latent ability space. In the unidimensional case, the general and restricted models become equivalent, with both simply reducing to the NRM and differing only in their parameterization.

**Figure 2:**
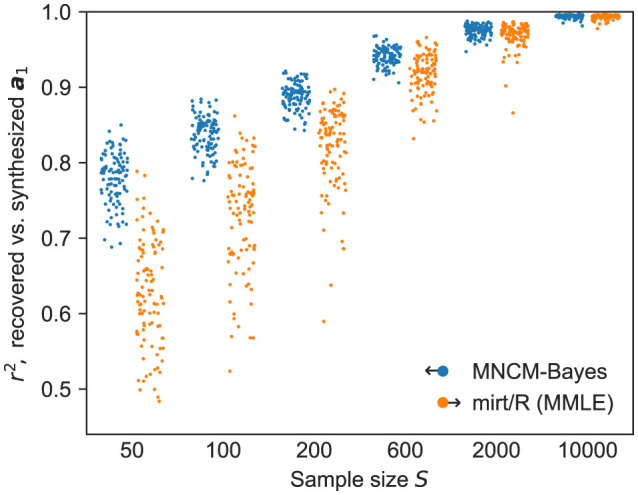
Coefficients of determination for recovered vs. synthesized slopes using unidimensional synthetic data, comparing MNCM-Bayes (blue, offset left) to established IRT software implementing MMLE (orange, offset right) with default settings. Points show results from individual replications, with jitter used to reduce overplotting.

As shown in Figure 2, the MNCM-Bayes procedure performs quite favorably in this comparison, especially at smaller sample sizes, with some improvements to robustness visible up to sample sizes of *S* = 2, 000. These results are somewhat expected given the lack of item-parameter priors in the benchmark method, but still provide additional confirmation of the correctness and utility of our implementation.

Nevertheless, these results do help underscore a key advantage of hierarchical priors: improved performance for novice users. Even when IRT packages do support the use of item priors, it is typically up to the user to manually configure the distributions and widths of these priors, and later evaluate the results to determine whether their choices were adequate. If too wide a prior distribution is used, some of the benefits of increased robustness will be lost. Too narrow a prior, on the other hand, may excessively bias parameter estimates by allowing insufficient flexibility to fit the observed data. Understandably, this requires more experience than simply fitting a model to data with the default options. In contrast, a hierarchical Bayesian model requires little or no manual tuning to achieve substantially-improved results, as the prior widths are themselves learned from the data.

Finally, an astute observer may note that MNCM-Bayes exhibits slightly better performance in Figure 2 compared to the results for *d* = 1 in Figure 1, especially at lower sample sizes. This is likely attributable to the model being better specified here: i.e., we are fitting a unidimensional model to a unidimensional dataset. In contrast, any synthesized dimensions left unextracted when fitting the multidimensional datasets serve as a source of unmodeled noise, which may reduce the method's accuracy when recovering the slopes in the remaining dimensions.

## 6 Real data example: the force concept inventory

To demonstrate the promise of our method in a more realistic application, we present some preliminary results from applying the MNCM-Bayes to data obtained from administrations of the Force Concept Inventory. While these results do not yet meet the standard of rigor required to serve as standalone research findings (which we hope to provide in a followup paper), they do show that the method can identify real misconceptions in real concept-test data.

The FCI, which grew out of work by David Hestenes' group in the mid-1980s, was first published by Hestenes et al. (1992) and later revised by Halloun et al. (1995). This popular assessment asked straightforward questions about simple physical situations that were covered in the introductory weeks of typical college-level Newtonian mechanics courses, but that were known from research to reveal student misconceptions (e.g., “what forces act on a ball that is thrown vertically upwards?”). College teachers predicted that their students would score very highly on this instrument and were doubly shocked. Not only did their students score a mere 55% post-instruction, but they had already scored just over 40% on the pretest: thus the teachers taught their students less than a quarter of the important mechanics concepts that they didn't already know on day one of the course.

Our FCI dataset comprises post-instruction responses from *S* = 10, 039 students at a state university in the Southwestern part of the United States. All administrations used the “v95” revised version of the instrument (Halloun et al., 1995). We restricted our analysis to students responding to all 30 questions on the test in the expectation that these students were more likely to respond thoughtfully, which excluded 433 students from the sample. The MNCM-Bayes method was configured to allow a maximum of 16 dimensions, and returned a 14-dimensional fit for this dataset.

### 6.1 Rotating results for interpretability

A key challenge that arises in applying multi-dimensional IRT models like the MNCM to real data is that of ascribing meaning to the dimensions thereby discovered. As with principal component analysis, the dimensions extracted by MNCM-Bayes are initially identified in a principal basis. Such solutions are generally not easy to interpret, as each dimension loads on many categories across many items.

The standard approach for increasing the interpretability of such results (both in multidimensional IRT and in classical factor analysis techniques) is to find a transformation which increases some measure of “simplicity” while not changing the modeled probabilities. Many such measures exist, as do standard methods for transforming solutions to maximize them given a matrix of slopes or factor loadings. For the sake of brevity, we will forgo a principled evaluation of these methods for now and present only one as an example: a bi-factor rotation method proposed by Jennrich and Bentler (2011) and implemented in the GPArotation R package (Bernaards and Jennrich, 2005).

Bi-factor rotation methods such as the one above transform the coordinate system of ***Θ*** and ***A*** such that each distractor has non-negligible slopes along only a small number of dimensions (ideally just two). The first of these dimensions is always a ‘general' factor on which all distractors load—we have found the ability in this dimension to be strongly associated with students' overall test score (Spearman rank correlation coefficient ρ_s_ = 0.98). The remainder are “group” factors associated with only a small set of distractors. We call the slope vectors for each of these *sparse distractor vectors*, and define their positive directions as those which yield a positive Spearman correlation between the corresponding rows of ***Θ*** and the test scores. (This sign determination method is sometimes marginal as some dimensions do not correlate strongly with score; for those that do, however, this causes the slope components which most characterize the non-Newtonian nature of each distractor to have negative signs).

**Figure 3:**
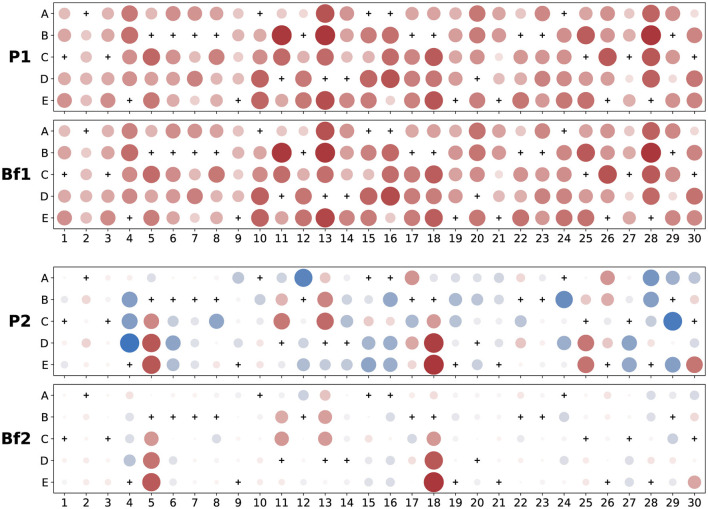
Examples of principal and sparse distractor vectors (${\hat{a}}_{d}$ coefficients) for FCI post-test data. Dots are colored red when negative (blue if positive) with size and intensity showing magnitude relative to the largest coefficient in a given panel; correct answer choices are marked with a “+” and always have zero slope due to model identification constraints. The top two vectors are from the first dimension—labeled “P1” for principal and “Bf1” for bi-factor rotated. They are correlated with each other (Pearson uncentered) at 0.998 and appear so visually similar that we carefully checked for accidental duplication. The second principal vector, “P2,” is obviously much denser than the second dimension of the rotated vector, “Bf2,” which has just a few large components that all deviate from zero in the negative (anti-Newtonian) direction.

To demonstrate the results of this transformation, we plot in Figure 3 the first two principal and sparse distractor vectors found for the large post-instruction data set described above. We display the components of each vector as a pattern of dots on an *I* × *C* grid, each dot having a size and intensity proportional to the magnitude of that component and being colored red when negative (blue if positive).

### 6.2 Some dimensions represent misconceptions

Each sparse distractor vector loads heavily on just a few distractors. To determine whether a particular vector represents a misconception, we examine the most heavily weighted distractors (typically the largest ~half-dozen assuming these stand out prominently from the background when plotted as in Figure 3) and see if selecting them would indicate consistent application of some alternate hypothesis to Newtonian mechanics. This process is admittedly quite subjective and could likely be improved upon in future work (e.g., by using outlier analysis to differentiate between prominent and background values), but nevertheless identifies several clear examples of misconceptions in our present results.

#### 6.2.1 Impetus force along curved path

As an example, consider the distractor vector labeled “Bf2” in Figure 3. Its six largest distractor components are on items 5 and 18, whose corresponding text is shown in Table 2. All six highly loaded distractors involve “force in the direction of motion.” Importantly, we note that the path is curved in both of these items, so we call this dimension *Impetus Force Along Curved Path*.

**Table 2:** Most heavily loaded response categories for second dimension of bi-factor rotated FCI post-test results (shown as “Bf2” in Figure 3). These choices are consistent with a student belief that an impetus force exists along curved paths.

**Item**	**Choice**	**Text**	**Slope $\hat{a}$**
***Forces on a ball traveling in a circular track***			
5	C	A force in the direction of motion	−1.19
	D	…and a centripetal force	−1.62
	E	…and a centrifugal force	−1.93
***Forces on a boy swinging on a swing***			
18	C	A force in the direction of the boy's motion	−1.18
	D	…and a centripetal force	−1.96
	E	…and a centrifugal force	−2.34

Impetus is best described as the Arabic and medieval physics concept that the force from the thrower imparts not only immediate motion to the projectile (as Aristotle said) but also a kind of “internalized force” that continues pushing it forward after it is no longer in contact with the thrower. We stress that this dimension applies impetus force to circular motion but less so to rectilinear motion as shown by the lesser loading on distractors 11B & C and 13B & C, which involve impetus force in linear motion.

#### 6.2.2 Last force determines motion

**Figure 4:**
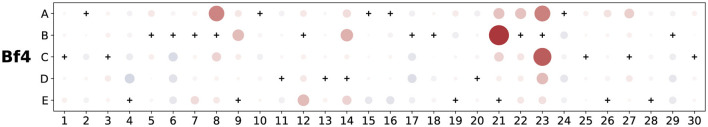
Fourth sparse distractor vector (“Bf4”) from bi-factor rotated FCI post-test results, encoding a *Latest Force Determines Motion* misconception. Dots are colored red when negative (blue if positive) with size and intensity showing relative magnitude; correct answer choices are marked with a “+.” Table 3 summarizes the dominant response choices.

Another example is distractor vector “Bf4” (i.e., the fourth dimension of the bi-factor rotated slopes), which is shown in Figure 4. The dominant components of this vector are described in Table 3.

**Table 3:** Most heavily loaded response categories for fourth dimension of bi-factor rotated FCI post-test results (shown in Figure 4).

**Item**	**Choice**	**Text**	**Slope $\hat{a}$**
***Puck moving along** *x* **is kicked by foot moving along** *y**			
8	A	Puck goes in direction of kick	−1.46
***Rocket moving along** *x* **pointing along** *y* **starts firing***			
21	B	Rocket goes straight along *y* axis	−2.44
***Rocket engine is now turned off***			
23	A	Rocket goes straight along *x* axis	−1.51
	C	Rocket goes straight along *y* axis	−1.97

This dimension is mostly aligned with the (known) misconception that when a new force is applied to a moving object, the direction of motion immediately aligns with that new force, ignoring inertia and motion from previously applied forces—described as the “last force to act determines motion” view by Hestenes and Jackson (2010) who identify categories 8A, 9B, 21B, and 23C as exemplars. This misconception is usually understood to include an expectation that the new motion persists at least initially after a force has ceased to act. Thus the inclusion in this misconception of 23A, where a rocket reverts to its *original* motion once its engine is turned off, is surprising and suggests further confusion about motion when forces *stop* being applied (a topic well outside the scope of this paper).

### 6.3 Summary of results

The limited results we just discussed are clear-cut examples showing that the combination of the MNCM-Bayes method followed by rotations to find sparse distractor vectors can discover known misconceptions—indeed, the two just discussed are among the top three found by Wheatley et al. (2022) using modified module analysis (Wells et al., 2019). Our results also illustrate potential improvements to our understanding of existing misconceptions, for example by showing that the impetus concept applies more strongly for motion in a curved path than in a straight path, or that the belief that only the last force applied determines an object's motion is also associated with a peculiar view of what happens when such forces stop acting.

The remainder of our results in this example application are included in the supplemental materials for this paper (both as figures and as tabulated slope coefficients). While several additional dimensions in these results seem to have precedents in prior misconceptions research, we postpone further discussion to a later application-focused paper with a more thorough analysis of our full FCI dataset (~34,000 exams, including pre- and post-instruction data from eight colleges and universities).

## 7 Concluding remarks

This work makes two primary contributions to the fields of psychometrics and education research:

1. We present a Bayesian approach to fitting the very general Multidimensional Nominal Categories Model which combines several recommendations in recent IRT literature—including the use of variational inference, hierarchical priors, and a fast approximate parameter initialization method. Using synthetic data, we explore the parameter recovery performance of our procedure, compare it to established IRT software in a limiting one-dimensional case, and demonstrate its robustness and self-limiting dimensionality behavior.
2. We present a fully-exploratory method aimed at discovering student misconceptions from multiple-choice concept test data. This method combines our general MNCM implementation with subsequent dimensional transformations to create sparse loadings which are usefully interpretable. Our findings suggest that this method is most likely to provide useful insights at large sample sizes (10,000+), and a real-data example provides preliminary evidence that this method can recover known misconceptions from student responses to the Force Concept Inventory, a pioneering research-developed concept test for Newtonian mechanics.

Overall, this synthesis of modern Bayesian methods with classical IRT and factor analysis techniques shows great promise for discovering student misconceptions from large sets of concept-test response data. While further work is needed to refine and validate our approach, we expect methods such as the one presented here to find broad applications in education, whether for conducting research on misconceptions, developing and refining concept inventories, or improving learning through targeted instruction.

### 7.1 Future work

Many opportunities exist for improving the MNCM-Bayes method or extending its capabilities. One that we are already investigating is the choice of factor rotation method, which must ideally balance ease of interpretation with consistency of the discovered misconceptions. By comparing results obtained using different rotations across data from several colleges, we hope to better inform this choice in future version of our method. Another modification would be to allow partially-specified patterns of loadings. This would allow us to manually associate some dimensions with particular known misconceptions (by constraining any irrelevant slopes to zero) and could aid in steering any unconstrained dimensions toward novel misconceptions instead of existing ones.

On the applications side, a compelling first question for future study is whether the various misconceptions that we found for the FCI are consistent across different colleges, and whether similar misconceptions are as robustly found in pre-instruction data rather than post-instruction data; we plan to address both topics in a forthcoming paper (in preparation). One could also study how student ability scores for each misconception depends on factors such as gender, preparation, or background. And, by comparing results from pre- and post-instruction data, one might determine which instructional approaches are most effective in reducing the persistence of various misconceptions.

As our methods are applied to other research-designed instruments, it seems likely that new misconceptions—or re-contextualized versions of existing ones—will be discovered, especially in subject areas where few studies of student misconceptions exist. Finally, we need not limit ourselves to data from traditional concept tests: modified versions of this method could be applied to entire online courses where frequently-given wrong answers have already been mined from student data (as is done by e.g., *MasteringPhysics.com* and *ExpertTA.com*) and could be treated as distractors. Further applications await.

## Data Availability

The data analyzed in this study is subject to the following licenses/restrictions: permission from originating institution(s) required to access. Requests to access these datasets should be directed to David Pritchard, dpritch@mit.edu.

## Footnotes

^1^Note, this use of *b* should not be conflated with the difficulty parameter in the two-parameter logistic IRT model; despite sharing a variable name, the two have distinctly different meanings. Many IRT sources avoid this conflict by instead using *c* for intercepts, though the use of *b* is not without precedent (cf. Takane and de Leeuw, 1987, e.q. 19). We argue that reserving “*c*” as an index variable for categories improves overall readability since the latter appears in formulas far more often than the former.

^2^In the context of an educational assessment, one would assume these “preference values” are derived from a student's perceived likelihood of each option being the correct response.

^3^The “learning rate” is the Adam analog of a step size in other gradient descent optimization methods.

^4^Specifically, we adapt “Algorithm 1” Zhang et al., (2020, p. 359–360) to work with nominal data by setting their inverse link function “*f*” to our Equation 1 and their input response matrix “***Y***” to an *S* × *IC* indicator matrix with components derived from our (nominal) matrix ***R*** according to

$$y_{s}^{\left(i,c\right)}=\{\begin{array}{ll} 1 & \text{if }r_{s}^{\left(i\right)}=c,\\ 0 & \text{otherwise}. \end{array}$$

The number of factors “*K*” and truncation parameter “ϵ” in Algorithm 1 are set to *D* and 10^−4^, respectively. The outputs of the algorithm serve as initial guesses for the posterior means of ***Θ***, ***A***, and ***b***, with the corresponding initial guesses for **α** and β computed by taking the standard deviations of ***A*** (separately for each dimension) and ***b***.

^5^This may be seen by factoring the inner product of ***Θ*** and ***A*** via compact singular value decomposition followed by some algebraic manipulation,

$$\begin{array}{l} \Theta A=U\Sigma V^{⊺}=\left(U\sqrt{S}\right)\left(\Sigma V^{⊺}\sqrt{S}\right)=\Theta^{⋆}A^{⋆}, \end{array}$$

where ***U*** ∈ ℝ^*S* × *D*^ and ***V***^⊺^ ∈ ℝ^*D* × *IC*^ are both semi-orthogonal and **Σ** ∈ ℝ^*D* × *D*^ is diagonal. The transformed variables $\Theta^{⋆}\equiv U\sqrt{S}$ and $A^{⋆}\equiv \Sigma V^{⊺}/\sqrt{S}$ then have diagonal covariance matrices Cov[***Θ***^⋆^] = ***I*** and Cov[***A***^⋆^] = **Σ**^2^/(*SIC*), with the entries of the latter conventionally arranged in descending order.

^6^These “unused” dimensions did still seem to contribute a small but consistent amount of noise to the computed tendencies, despite the fact that an additive noise term should not be necessary in a logistic model like the MNCM. We have not yet determined whether this behavior reflects the “true” posterior distribution of the model parameters or is merely an artifact arising from our use of approximate inference techniques.
