# Supplementary figures and images for: A multidimensional Bayesian IRT method for discovering misconceptions from concept test data
Source: Front Psychol. 2025 Jan 29;16:1506320. doi: 10.3389/fpsyg.2025.1506320 (PMC11841472; doi:10.3389/fpsyg.2025.1506320)

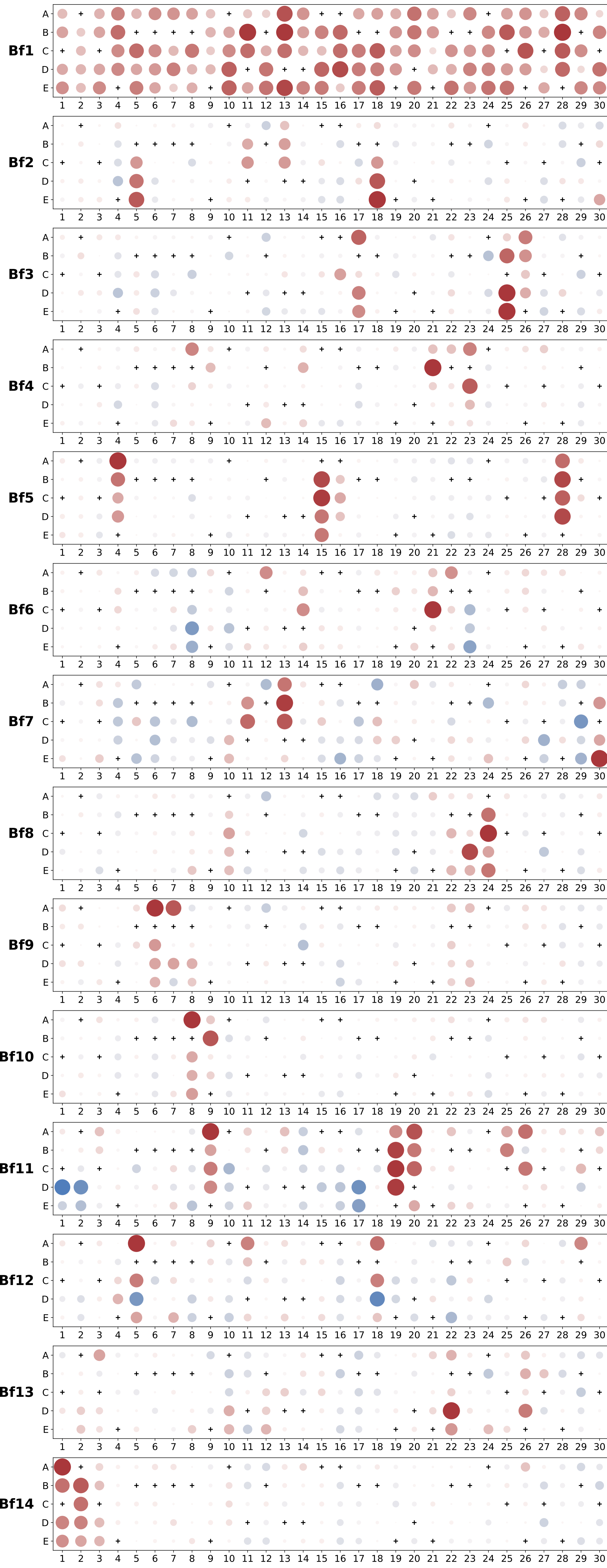

Supplement: Supplementary file 2 [file Data_Sheet_2.pdf]
